# Hypobaric hypoxia induced renal injury in rats: Prophylactic amelioration by quercetin supplementation

**DOI:** 10.1371/journal.pone.0279304

**Published:** 2023-02-24

**Authors:** Vaishnavi Rathi, Isha Tiwari, Ritu Kulshreshtha, Sarada S. K. Sagi

**Affiliations:** 1 Defence Institute of Physiology and Allied Sciences, DRDO, Delhi, India; 2 Vallabhbhai Patel Chest Institute, University of Delhi, New Delhi, India; Zagazig University, EGYPT

## Abstract

The present study aims at assessing the effect of hypobaric hypoxia induced renal damage and associated renal functions in male SD rats. Further, this study was extended to explore the protective efficacy of quercetin in ameliorating the functional impairment in kidneys of rats under hypobaric hypoxia. Rats were exposed to 7620m (25000 ft.) at 25°C ±2 in a simulated hypobaric hypoxia chamber for different time durations (0h,1h, 3h, 6h, 12h, 24h and 48h) in order to optimize the time at which maximum renal damage would occur. The rats were exposed to hypoxia for 12h duration was considered as the optimum time, due to significant increase in oxidative stress (ROS, MDA) and renal metabolites (creatinine, BUN and uric acid) with remarkable reduction (p<0.001) in antioxidants (GSH) in plasma, as compared to other tested durations. Moreover, these findings were in support with the histopathology analysis of renal tissues. For optimum quercetin dose selection, the rats were administered with different doses of quercetin (25mg, 50mg, 100mg and 200mg/Kg BW) for 12h at 7620 m, 25°C ±2, 1h prior to hypoxia exposure. Quercetin 50mg/kg BW was considered as the optimum dose at which significant (p<0.001) reduction in oxidative stress levels followed by reduction in creatinine and BUN levels were obtained in plasma of the rats compared to hypoxia control rats. Quercetin prophylaxis (50mg/kg BW) stabilized the HIF-1α protein expression followed by reduced VEGF protein expression along with reduced levels of LDH (p<0.001) in the kidneys of rats compared to hypoxia control. Histopathological observations further substantiated these findings in reducing the renal tissue injury. The study findings revealed that, quercetin prophylaxis abrogates the possibility of hypobaric hypoxia induced renal injury by reducing the oxidative stress in rats.

## Introduction

Maladaptive response to high altitude hypoxia exposure occurs in unacclimatized individuals that reflect into life-threatening complications viz. Acute mountain sickness (AMS), High Altitude Cerebral Edema (HACE) and High Altitude Pulmonary Edema (HAPE) [1, 2]. Consequently, all these three ailments may exist in combination or alone. However, people ascending to high altitude also manifest renal dysfunctions characterized by microalbuminuria, polycythemia, hyper uricemia with preserved glomerular filtration rate (GFR) together termed as High Altitude Renal Syndrome (HARS) [3, 4]. The renal system regulates acid-base balance, electrolytes, and innumerable hormones along with hyperventilation thus plays a crucial role in acclimatization and high-altitude illnesses [5]. In addition, rapid and unexplained diuresis at high altitude indicate towards the role of kidneys as early blood/ tissue oxygen sensors [6, 7]. Conventionally, the physiological response to acute hypobaric hypoxia aims at elevating oxygen supply to tissues by increasing ventilation, cardiac output, hemoglobin concentration, and respiratory alkalosis, etc. However, kidneys compensate for alkalosis by preserving hydrogen ions and excreting excess bicarbonate ions thus increasing erythropoiesis which leads to improved oxygen delivery via increased hemoglobin and angiogenesis [8].

Renal oxygenation (pO_2_) is maintained in a narrow range by the interplay of arterial oxygen content, arteriovenous O_2_ shunting (results from counter-current exchange of oxygen among arterial and venous vessels before arterial blood reaches renal microcirculation) and oxygen consumption (VO_2_). Though, kidney receives 20% of cardiac output, measured pO_2_ is surprisingly as low as 5–20 mmHg in medulla and 50-60mmHg in cortex which contribute towards its greater susceptibility to hypoxia associated renal pathologies [9, 10]. Moreover, abundance of polyunsaturated fatty acids (PUFA) along with excessive reactive oxygen species (ROS) production due to soluble molecular oxygen in kidneys makes them vulnerable to be attacked by free radicles [11]. In addition, PUFAs convert themselves into reactive free radicals via lipid peroxidation chain reaction when attacked by super-oxides, hydroxyl ions etc. that renders kidneys imprudent to oxidative stress [12–14]. Due to constantly functioning in low pO_2_ environment, renal cells have evolved a variety of molecular mechanisms that allow them to adapt and respond efficiently to low oxygen tension. Apart from a number of transcription factors involved in cellular adaptation to hypoxia, hypoxia inducible factors (HIFs) are the key regulatory players having therapeutic as well as Reno protective potential. Under normoxia conditions, hydroxylation of HIF-1α is attained via interactions with oxygen sensing, prolyl hydroxylase domain (PHDs) protein, which is further targeted by von Hippel-Lindau-E3 ubiquitin ligase complex for proteasomal degradation. However, under hypoxia, hydroxylation is inhibited and HIF signaling is activated thus it’s binding with hundreds of target genes initiate transcription of VEGF, erythropoietin etc. [15]. Oxygen-dependent, catalytic activity of HIF hydroxylases is also mediated by reactive oxygen species (ROS) and nitic oxide (NO) linking various intracellular signaling pathways [16, 17].

Rodrigo and Rivera (2002) and Zager et al., (2013) have reported the importance of involvement of ROS and lactate dehydrogenase (LDH) in causing oxidant injury in renal tissues [18, 19]. Oxidative stress mediated renal injury leads to glomerular, tubule-interstitial and endothelial alterations. Moreover, the glomerulus is considerably more sensitive to oxidative stress than other segments of nephron and also aggravation in endothelial and mesangial cells in kidneys result in its functional and structural impairment [18, 19].

In view of the above-mentioned ROS mediated injuries, interventions favoring scavenging or depuration of ROS should attenuate or prevent the oxidative stress, thereby mitigating against subsequent renal damage. Hence, pretreatment with quercetin, an anti-oxidative polyphenol should result in relevant glomerular and renal protective therapy. Protective role of quercetin in ameliorating the vascular leakage in lungs and brain of rats under hypobaric hypoxia exposure have already been reported earlier in the literature [20, 21].

Despite having a vast knowledge of high altitude physiology and illnesses, our understanding for functioning of renal system in hypobaric hypoxia environment is scarce and corresponding studies on animal model are insufficient. Thus, the present study aims at assessing the effect of hypobaric hypoxia induced ROS on renal system by determining the renal function parameters and tissue injury marker in rats. The study is designed into three phases: (i) time duration optimization at which maximum renal damage is occurring under hypobaric hypoxia (ii) quercetin dose optimization study under hypobaric hypoxia and (iii) exploring the protective efficacy of quercetin in prevention of oxidative stress induced renal injuries in rats under hypobaric hypoxia.

## Material and methods

### Chemicals and reagents

Quercetin and dichlorofluroscein sodium salt were procured from Sigma Aldrich (St. Louis MO, USA), Dimethylsulphoxide (DMSO) from Sisco Research Laboratory (SRL, Maharashtra) along with Thiobarbituric acid (TBA) and Tricarboxylic acid (TCA). 5’5’-dithio-bis-(2- nitro-benzoic acid) (DTNB) from Sigma Aldrich. All the other chemicals and reagents were of analytical grade.

### Drug preparation

Quercetin was freshly prepared in 0.5% DMSO and supplemented orally to animals 1h prior to hypobaric hypoxia exposure in simulated hypobaric hypoxia chamber.

### Experimental animals

Male Sprague Dawley (SD) rats of weight 180-200g were obtained from central animal facility of DIPAS-DRDO, Delhi, India. Animals were kept in experimentally designed polypropylene cages of dimension 32in. ×24in. ×16in. provided with standard conditions (12 light/dark cycle, 25± 2°C temperature and 55±5% relative humidity) and availability of animal chow and water *ad libitum*. All the animal studies and protocols are in accordance with standards provided in the guide for the Care and Use of Laboratory Animals (National Academy of Sciences, Washington, DC). Protocols involving animal studies were reviewed and sanctioned by the Institutional Animal Ethics Committee (IAEC), DIPAS, Delhi, India, accredited to Committee for the Purpose of Control and Supervision of Experiments on Animals (CPCSEA), Government of India.

### Experimental protocols

The study was carried out in three phases.

#### Phase 1 (Time duration optimization study)

Phase 1 studies were performed to optimize the time duration at which maximum renal damage occurs in rats exposed to hypobaric hypoxia. Hence, animals were exposed at 7620m (25,000 ft.) for different time durations in hours (h) i.e. 0h (normoxia control), 1h, 3h, 6h, 12h, 24h & 48h (each group containing 6 rats) at 25± 2°C in a simulated hypobaric hypoxia chamber.

#### Phase II (Dose optimization study)

This phase was designed to determine the optimal dosage of quercetin required to minimize the renal damage induced by hypobaric hypoxia. This includes administration of different doses of quercetin i.e. 25, 50, 100 and 200mg/kg BW 1h prior to hypoxia exposure for 12h (optimum hypobaric hypoxia exposure duration determined from the Phase 1 results). All groups including control contained 6 animals each.

#### Phase III

Phase -II studies confirmed that the effective minimization of oxidative stress and renal damage was achieved by Quercetin supplementation at 50mg/kg BW as compared to other tested doses in rats under hypobaric hypoxia. Thus, the remaining study (Phase-III) was carried out using this dose of quercetin. Phase-III study was performed on 4 groups of SD rats with each group containing 6 animals. The 4 groups include: (i) Normoxia control that received only vehicle (ii) Hypoxia control that received only vehicle 1h prior to hypobaric hypoxia (12h) (iii) Control + Quercetin group was supplemented with 50mg/kg BW without hypoxia exposure and (iv) Hypoxia + Quercetin group was supplemented with 50mg/kg BW 1h prior to hypoxia exposure of 12hr.

### Hypoxia exposure

Animals were exposed in a simulated hypobaric hypoxia chamber (Matrix, India) for different durations (viz. 0h, 1h, 3h, 6h, 12h, 24h and 48h) at an altitude of 7620m (25000 ft.), 25°C ±2. The chamber core pressure was maintained around 280 mm Hg (equivalent to 8% O_2_ concentration). Fresh air was flushed at the rate of 4 lit/h along with the relative humidity at 55 ± 5% inside the hypoxia chamber. Further, the partial pressure of oxygen (PO_2_) in normoxia control rats was observed to be around 96 ±2 mmHg, whereas in hypoxia exposed rats PO_2_ was around 35±2 mmHg indicated that, the animals were exposed to low barometric pressure at high altitude. Standard animal chow and water was made available *ad libitum* to rats during hypoxia exposure. All animal experiments were performed with utmost care to minimize the sufferings on rats.

### Biochemical parameters

#### Method of sacrifice

Both normoxia rats and the rats exposed to different hours of hypoxia, supplemented with different doses of quercetin were sacrificed using Ketamine-Xylazine cocktail (80:20 mg/kg BW ratio) given intraperitoneally as an anesthesia.

#### Sample preparation

Animals were perfused with chilled 1X PBS at the time of dissection. Kidney tissues were washed with 1X PBS and homogenized (10%) in 0.154 M KCl containing DTT, PMSF and protease inhibitor cocktail (PIC) for analyzing the biochemical estimations. Blood was collected from left ventricle of the heart, plasma was separated and kept at -80°C for further biochemical analysis.

#### Measurement of oxidative stress

Estimation of reactive oxygen species (ROS) generation in kidney homogenate was carried out using 2, 7-dichlorofluorescein diacetate (DCF-DA) assay in normoxia and hypoxia exposed rats [22]. DCFH-DA along with potassium dihydrogen buffer was added to tissue homogenates prior to incubation for 15 min in dark at room temperature (RT). Fluorescence emitted by DCF formed as a result of oxidation of DCFH-DA in the presence of ROS, was measured spectrophotometrically (Synergy H1, Biotek, Germany) at an excitation of 485 nm and emission of 530 nm.

#### Malondialdedhyde (MDA) estimation

The assay involves condensation reaction of two molecules of TBA with one molecule of MDA which is formed as a byproduct of lipid peroxidation in tissue homogenates of all normoxia, hypoxia exposed and quercetin supplemented groups. The method consists of heating -up the assay mixture consisting of tissue (kidney) homogenates with TBA, TCA and HCl in a boiling water bath (80°C) for 1h. Later, the assay reaction mixture was allowed to cool at Room temperature (RT) and then centrifuged at 3000 rpm for 10min at 4°C. The absorbance of the MDA-TBA adduct formed (supernatant) was measured spectrophotometrically at 532 nm [23, 24].

#### Determination of reduced glutathione (GSH)

The kidney tissue homogenates of all the tested groups (normoxia, hypobaric hypoxia exposed and quercetin supplemented groups) were precipitated with precipitating reagent, incubated for 5min at RT and further centrifuged at 1200xg for 20 min at 4°c. The supernatant obtained was mixed thoroughly with phosphate buffer and 5,5-Dithiobis (2-nitrobenzoic acid) (DTNB) reagent. The color obtained was measured spectrophotometrically at 412 nm [25, 26].

#### Measurement of creatinine

Creatinine is a muscle metabolic waste, eliminated by kidneys with urine regularly. Plasma creatinine was measured to assay the functioning state of kidneys. The creatinine in plasma of normoxia, hypobaric hypoxia exposed and quercetin supplemented groups were quantified by using creatinine assay kit (Synergy Bio) based on Jaffe’s method [27].

#### Quantification of Blood Urea Nitrogen (BUN)

The assay was carried out in plasma of normoxia, hypoxia exposed and quercetin supplemented groups to quantify the urea nitrogen, a waste product released by the liver. The method was performed in accordance with the manufacturer’s instructions (Synergy Bio).

#### Estimation of Lactate Dehydrogenase (LDH)

Lactate dehydrogenase (LDH) is a crucial indicator of tissue injury and cytotoxicity. The LDH activity in the kidneys of the rats (normoxia, hypobaric hypoxia exposed and quercetin supplemented groups) was measured by using commercial kit provided by Bio Assay systems (D2DH-100) following the procedure mentioned in the manufacture’s guidelines.

#### Estimation of uric acid

Uric acid is a body waste product formed due to breakdown of purines and one of the markers for assessing of Kidney functions. It is a regular eliminatory product from the body. The plasma levels of uric acid from normoxia, hypobaric hypoxia exposed and quercetin supplemented groups was estimated using commercially available uric acid kit provided by Synergy bio (Uricase method) as per manufacturer’s instructions.

### Protein expression studies

Cytoplasmic and nuclear extracts were prepared using lysis (10 mM HEPES, 1.5 mM MgCl_2_, 10 mM KCl) and extraction buffers (20 mM HEPES, 1.5 mM MgCl_2_, 0.42 M NaCl, 0.2 mM EDTA, 25% glycerol). Protein quantification was estimated by Lowry’s method (1951) [28]. Western blot analysis was carried out for separation and identification of hypoxia inducible factor-1alpha (HIF-1α) and one of its associated gene vascular endothelial growth factor (VEGF) and β-actin in kidneys of rats exposed to hypoxia. The protein in kidney samples were separated using 10% SDS-PAGE. The separated proteins were then, electro-blotted to the nitrocellulose membranes and blocked with 5% BSA dissolved in 1X PBST (pH 7.4) at RT for 1h with gentle shaking. Membranes were then washed and incubated with respective primary antibodies (Santa Cruz Biotechnology (HIF-1α), Invitrogen (VEGF); 1: 1000 dilution) over night at 4°C. After 4–5 washings with PBST (Tween 0.1%), the membranes were probed with HRP-conjugated secondary antibodies (Santa Cruz Biotechnology 1: 15000 dilution) at RT for 2h. The blots were then thoroughly washed (4–5 times) with PBST. The membranes were developed using chemiluminescent peroxidase substrate (Luminata forte, Millipore U.S.A) and the bands were visualized in Chemidoc (UVP, Cambridge, U.K). The optical density (OD) of the bands were quantified using lab works software (UVP-Bio Imaging systems, CA).

### Histopathological examination

Histopathological examination was carried out by staining the kidney sections of normoxia, hypobaric hypoxia exposed and quercetin supplemented groups with hematoxylin & eosin (H & E staining) stain. For histopathological slides preparation, after anesthesia all the experimental and control group animals were perfused with 4% paraformaldehyde. Kidneys were then, immediately extracted and again immersed in 4% paraformaldehyde. The tissues were then, truncated into fine sections of 5μm thickness and stained with hematoxylin and eosin. Finally, photomicrographic images were captured using Nikon 901 microscope (Nikon, Japan).

### Statistical analysis

The results obtained were statistically analyzed using Graph Pad Prism software, California, U.S.A. Comparisons between experimental groups from all three phases of the study were made using one-way analysis of variance (ANOVA). Results were expressed as mean ±SD. Comparison between multiple groups were analyzed by One Way ANOVA followed by Bonferroni’s multiple comparison and unpaired t-test was used to compare the data between the two groups. Differences were considered statistically significant for p<0.05.

## Results

### Phase I: Optimization of hypobaric hypoxia exposure time duration study

#### Effect of hypobaric hypoxia exposure on oxidative stress parameters

**Fig 1:**
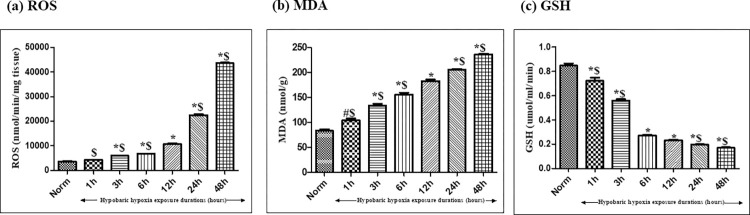
Effect of hypobaric hypoxia exposure on oxidative stress parameters in kidneys of rats exposed to 7620 m (25,000 ft.) at 25 ± 2°C for different time durations (i.e. Normoxia control (0h), 1h, 3h, 6h, 12h, 24h and 48h). (a) Reactive Oxygen Species (ROS) (b) Malondialdehyde (MDA) and (c) Reduced Glutathione (GSH). Values are mean ± SD (n = 6). (a) ROS: *p<0.001 Norm v/s hypobaric hypoxia exposures 3h, 6h,12h, 24h and 48h; $ p<0.001 hypobaric hypoxia exposure 12h v/s hypobaric hypoxia exposures 1h, 3h, 6h, 24h and 48h (b) MDA: # p<0.01 Norm v/s hypobaric hypoxia exposure 1h; *p<0.001 Norm v/s hypobaric hypoxia exposures 3h, 6h,12h, 24h and 48h; $ p<0.001 hypobaric hypoxia exposure 12h v/s hypobaric hypoxia exposure 1h, 3h, 6h, 24h and 48h (c) GSH: *p<0.001 Norm v/s hypobaric hypoxia exposures 1h, 3h, 6h,12h, 24h and 48h; $ p<0.001 hypoxia exposure 12h v/s hypoxia exposure 1h, 3h, 24h and 48h. Norm-Normoxia control.

*Reactive Oxygen Species (ROS)*. A minute but non-significant increase in ROS production was observed in 1h of hypobaric hypoxia exposure as compared to normoxia control rats (0h). Later, the increase in hypobaric hypoxia exposure durations from 3h to 48h, showed a consistent and a significant increase (p<0.001) in ROS production in kidneys of rats as compared to normoxia control. Among all the time durations tested, we found that, almost 2.8-fold (↑) significant increase (p< 0.001) was observed in ROS production in kidney of rats exposed for 12h as compared to normoxia control. It was also noticed that, the increase in ROS production was 2.2 fold (↑) in 24h and 4.1-fold (↑) in 48h exposure indicating the excessive generation of ROS compared to 12h of hypoxia exposure (Fig 1A).

*Lipid peroxidation (MDA)*. An uninterrupted, consistent and significant upregulation in MDA levels were observed in kidney tissues of rats exposed to increasing time duration of hypobaric hypoxia i.e. right from 1h (p<0.05) to 48h exposure (p<0.001) as compared to control (Fig 1B).

*Reduced glutathione (GSH)*. As a consequence of hypobaric hypoxia exposure, antioxidant system that includes GSH, experienced a downfall with the rising time duration of hypoxia exposure in kidney tissues of rats compared to normoxia control. A remarkable reduction was observed since the beginning of hypobaric hypoxia exposures i.e. from 1h hypobaric hypoxia exposure onwards that was continued with a similar pattern up to 48h of hypobaric hypoxia exposure in comparison to normoxia control rats. Noticeably, reduction corresponding to the animal group exposed to 12h of hypobaric hypoxia as compared to normoxia control group was speculated to be of 3.6 fold (↓) decrease in GSH levels that remained continued with the increasing time duration of hypobaric hypoxia exposures up to 24h and 48h (Fig 1C).

#### Effect of hypobaric hypoxia exposure on renal function parameters

**Fig 2:**
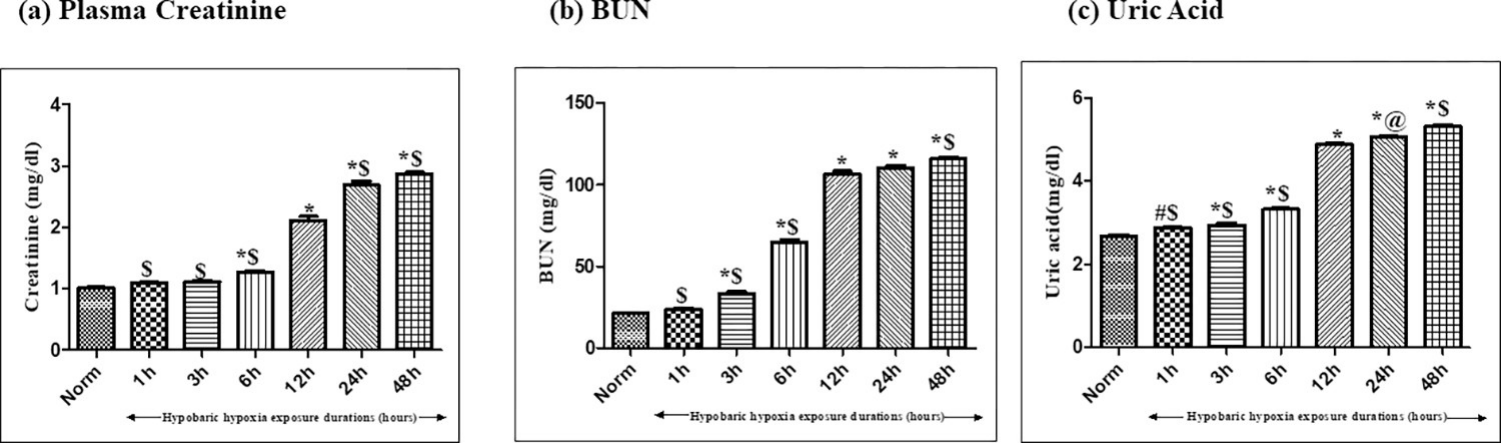
Effect of hypobaric hypoxia exposure on renal function parameters in rats exposed to 7620 m (25,000 ft.) at 25 ± 2°C for different time durations (i.e. Normoxia control (0h), 1h, 3h, 6h, 12h, 24h and 48h). Values are mean ± SD (n = 6). (a) Plasma Creatinine *p< 0.001 Norm v/s hypobaric hypoxia exposures 6h, 12h, 24h and 48h; ^$^p<0.001 hypobaric hypoxia exposure 12h v/s 1h, 3h, 6h, 24h and 48h (b) Blood Urea Nitrogen (BUN) *p< 0.001 Norm v/s hypobaric hypoxia exposures 3h, 6h, 12h, 24h and 48h; ^$^p<0.001 hypobaric hypoxia exposure 12h vs 1h, 3h, 6h and 48h (c) Uric Acid *p< 0.001 normoxia control v/s hypobaric hypoxia exposures 3h, 6h, 12h, 24h and 48h; #p< 0.05 normoxia control v/s 1h hypobaric hypoxia exposure; ^$^p<0.001 hypobaric hypoxia exposure 12h vs 1h, 3h, 6h and 48h, @p<0.05 hypobaric hypoxia exposure 12h v/s hypobaric hypoxia exposures 24h. Norm- Normoxia control.

*Creatinine*. Creatinine is a well-known breakdown product of creatine phosphate from muscle and protein metabolism. The results of the present study showed a significant and continuous increment with different duration of hypoxia exposure that indicate towards the impaired functioning of kidneys in low oxygen conditions. Exposure of rats to 1h and 3h of hypobaric hypoxia exposure failed to obtain a significant rise in creatinine levels. Noteworthy, a significant (p<0.05) rise in plasma creatinine levels was observed in 6h of hypobaric hypoxia exposure in correlation with normoxia control rats. Furthermore, an abrupt escalation (1.6 fold ↑) in plasma creatinine levels was perceived in rats exposed to 12h of hypobaric hypoxia as compared to 6h hypobaric hypoxia exposed rats. However, we have also observed that, as the time duration of hypobaric hypoxia exposure increased from 12h to 24h and further increased up to 48h, the creatinine levels remained more or less same; which indicated towards constant and tenacious damage caused by hypobaric hypoxia (Fig 2A).

*Blood Urea Nitrogen (BUN)*. An inappreciable and nonsignificant increment in BUN levels were seen in 1h of hypobaric hypoxia exposure as compared to the normoxia control group. However, 3h of hypobaric hypoxia exposure exhibited increased plasma BUN levels (3.1fold ↑) that signify, compromised renal functionality. Further, an upward trend was observed in subsequent hypobaric hypoxia exposure time durations i.e. 6h, 12h, 24h and 48h. A significant (nearly 4.9 fold ↑) retention of blood urea nitrogen was speculated in plasma of rats exposed to 12h of time duration in correlation with the normoxia control rats. Additionally, an abrupt increase in BUN levels were observed between 6h of hypobaric hypoxia exposure to 12h of hypobaric hypoxia exposure and further increase in durations (increased from 12h to 48h of hypobaric hypoxia exposure) exhibited a consistent increase in BUN levels with more or less same values, which indicates that, 12h time duration to be the optimum time at which maximum renal damage had occurred (Fig 2B). Although, significant rise was observed in 24 h and 48h of hypobaric hypoxia exposure too in comparison with the normoxia control rats (p<0.001) but the increment was nonsignificant when compared with 12h of hypobaric hypoxia exposure.

*Uric acid*. The results showed a significant (p<0.05) increment in uric acid levels in rats exposed to 1h and 3h of hypobaric hypoxia, however, the levels were observed to be prominently increased as the exposure duration of hypobaric hypoxia was raised up to 6 hours as compared to normoxia control. Further, the uric acid levels experienced a drastic upsurge as the hypobaric hypoxia exposure was enhanced up to 12h (1.8 fold ↑) as compared with normoxia control (p<0.001). Moreover, extending the hypobaric hypoxia exposure durations up to 24h and 48h, showed an increase in the uric acid levels which were nearly more or less similar to that of 12h of hypobaric hypoxia exposed group of rats (Fig 2C).

Thus, taken in to consideration of oxidative stress parameters and the kidney function tests, 12h was considered as the optimum time duration of hypobaric hypoxia exposure at which maximum renal damage had occurred in this study.

#### Effect of hypobaric hypoxia exposure on Lactate Dehydrogenase activity (LDH)

**Fig 3:**
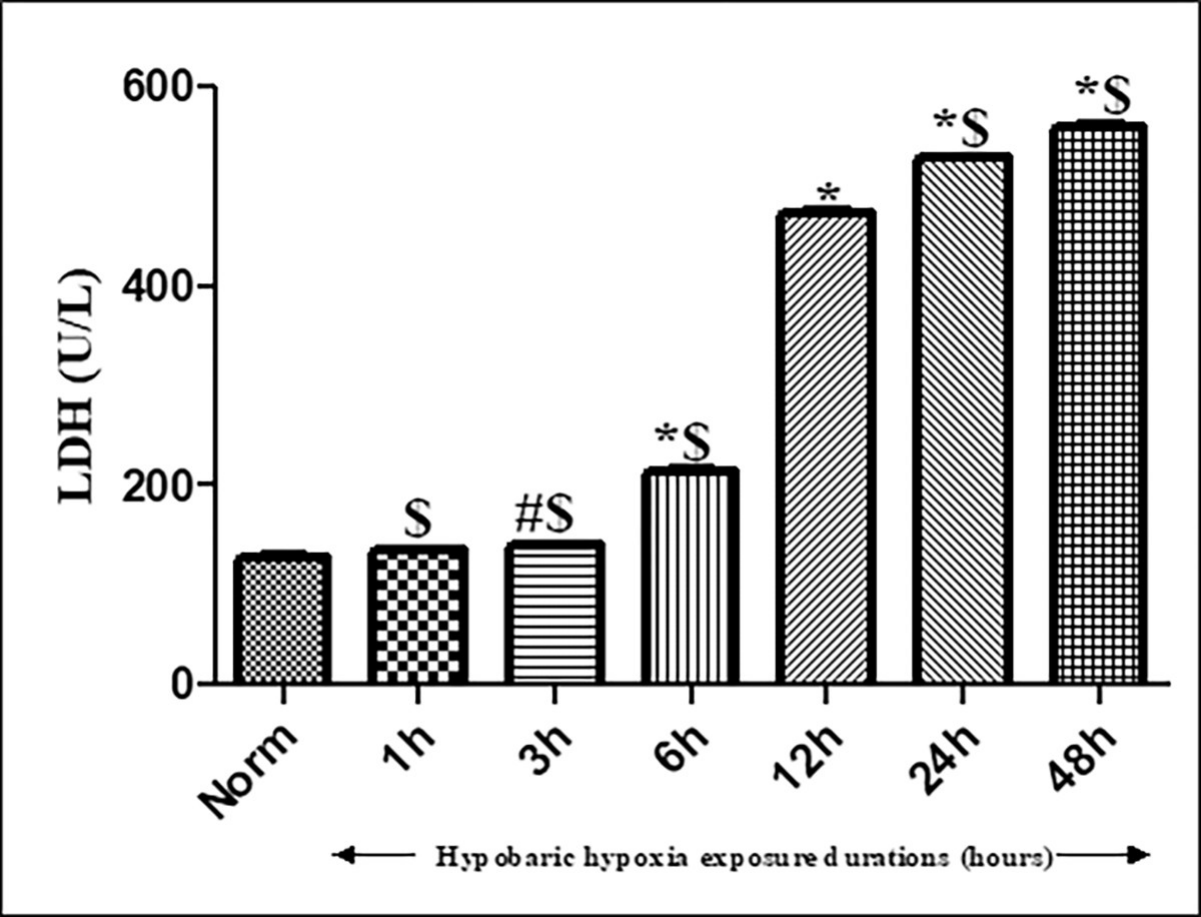
Effect of hypobaric hypoxia on Lactate Dehydrogenase activity (LDH) in rats exposed to hypobaric hypoxia of 7620m (25,000 ft.) at 25 ± 2°C for different time durations (i.e.1h, 3h, 6h, 12h, 24h and 48h). Values are mean ± SD (n = 6). *p< 0.001 Norm v/s hypobaric hypoxia exposures 6h, 12h, 24h and 48h; #p< 0.01 Norm v/s hypobaric hypoxia exposure 3h. ^$^p<0.001 hypobaric hypoxia exposures 12h vs 1h, 3h, 6h, 24h and 48 h. Norm-Normoxia control.

Lactate dehydrogenase (LDH) was employed as a marker for tissue injury and cytotoxicity. The results demonstrated that, no significant changes in LDH levels were observed in kidneys of rats exposed to 1h of hypobaric hypoxia as compared to normoxia control. However, significant difference in LDH levels were observed, when rats were exposed for 3h (p< 0.05) and 6h (p< 0.001) of hypobaric hypoxia as compared to control (Fig 3). However, further increase in time duration of the hypobaric hypoxia exposures from 6h to 12h showed a drastic upsurge in LDH levels as compared to normoxia control. Further rise in hypobaric hypoxia exposure durations (24 h and 48h) showed a significant increase (p<0.001) in LDH levels as compared to normoxia control; but showed more or less changes in comparison with 12h of hypobaric hypoxia exposure time duration, which is an indication of maximum renal damage started occurring from 12h of hypobaric hypoxia exposure compared among the other tested groups (Fig 3).

#### Effect of hypobaric hypoxia on protein expressions of HIF-1 α and VEGF

**Fig 4:**
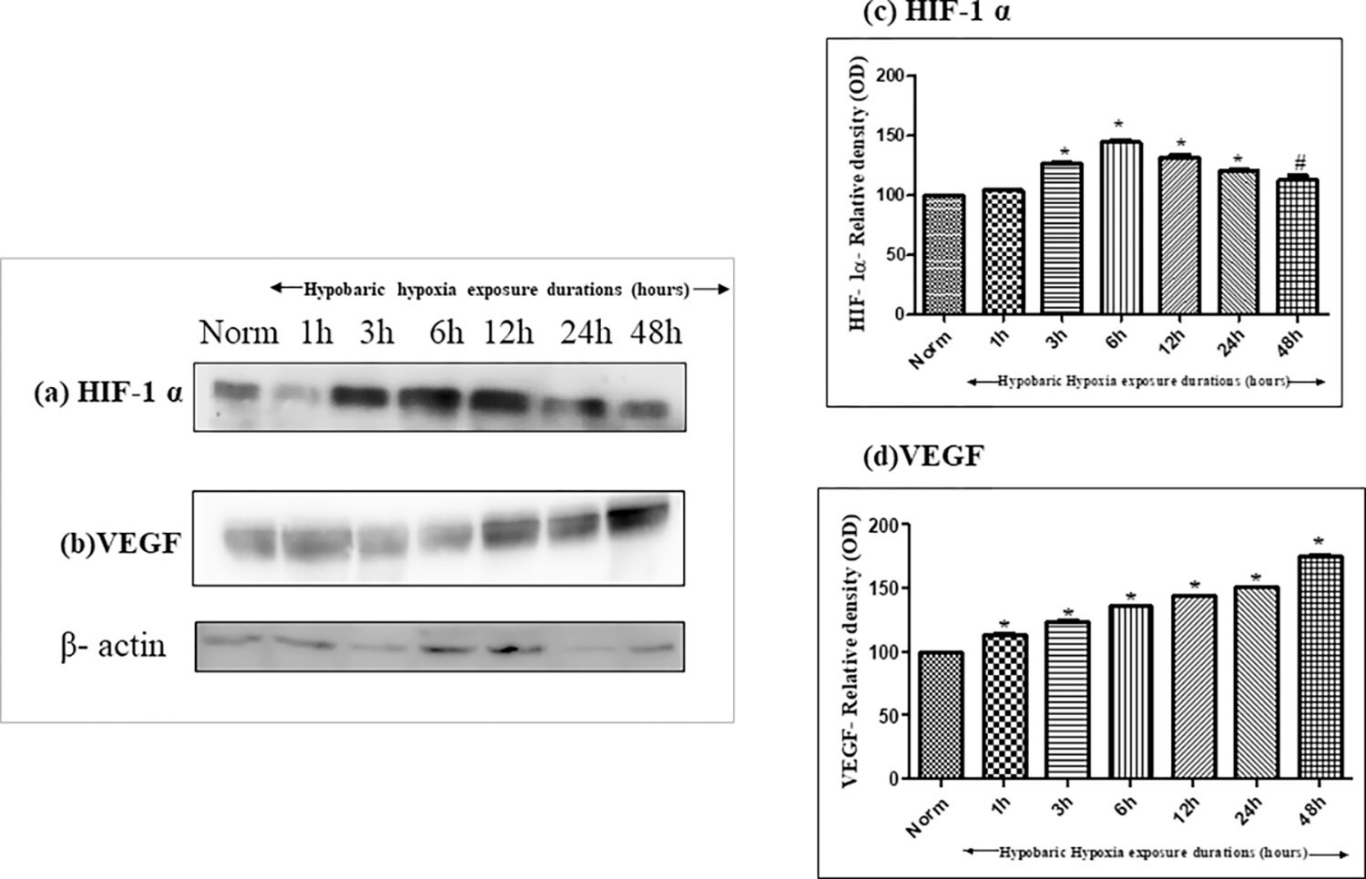
Effect of hypobaric hypoxia on protein expressions of (a) Hypoxia inducible factor- 1 α (HIF-1 α) and (b) Vascular Endothelial Growth Factor (VEGF) in kidneys of rats exposed to different hours of hypobaric hypoxia (1h, 3h, 6h, 12h, 24h and 48h) at 7620 m (25,000 ft.) at 25 ± 2°C. (c) Densitometry analysis of HIF-1 α: *p< 0.001 Norm v/s Hypobaric Hypoxia exposures 3h, 6h, 12h and 24h, #p<0.01 Norm vs hypobaric hypoxia 48h (d) Densitometry analysis of VEGF: *p< 0.001 Norm v/s hypobaric hypoxia exposures 1h, 3h 6h, 12h, 24h and 48h. Norm- Normoxia control.

Protein expressions of hypoxia inducible factor- 1α (HIF-1 α) (Fig 4A) and vascular endothelial growth factor (VEGF) (Fig 4B) were analyzed in kidney homogenates of hypobaric hypoxia exposed rats for different time durations. As the time duration of hypobaric hypoxia exposure increased, the HIF-1 α protein expression continued to increase, although a decline was observed after 12h of hypobaric hypoxic exposure, however, the expression was observed to be more than normoxia control, indicating the stabilization of HIF-1 α which is a common phenomenon at high altitude regions due to hypoxia prevalence. However, expression of VEGF protein was seen to be increased right from 1h of hypobaric hypoxia exposure onwards to 48h of hypobaric hypoxia exposure as compared to normoxia control. The densitometry analyses of these proteins were expressed in adjacent to their respective figures (Fig 4C for HIF-1 α and Fig 4D for VEGF).

#### Histopathological analysis

**Fig 5:**
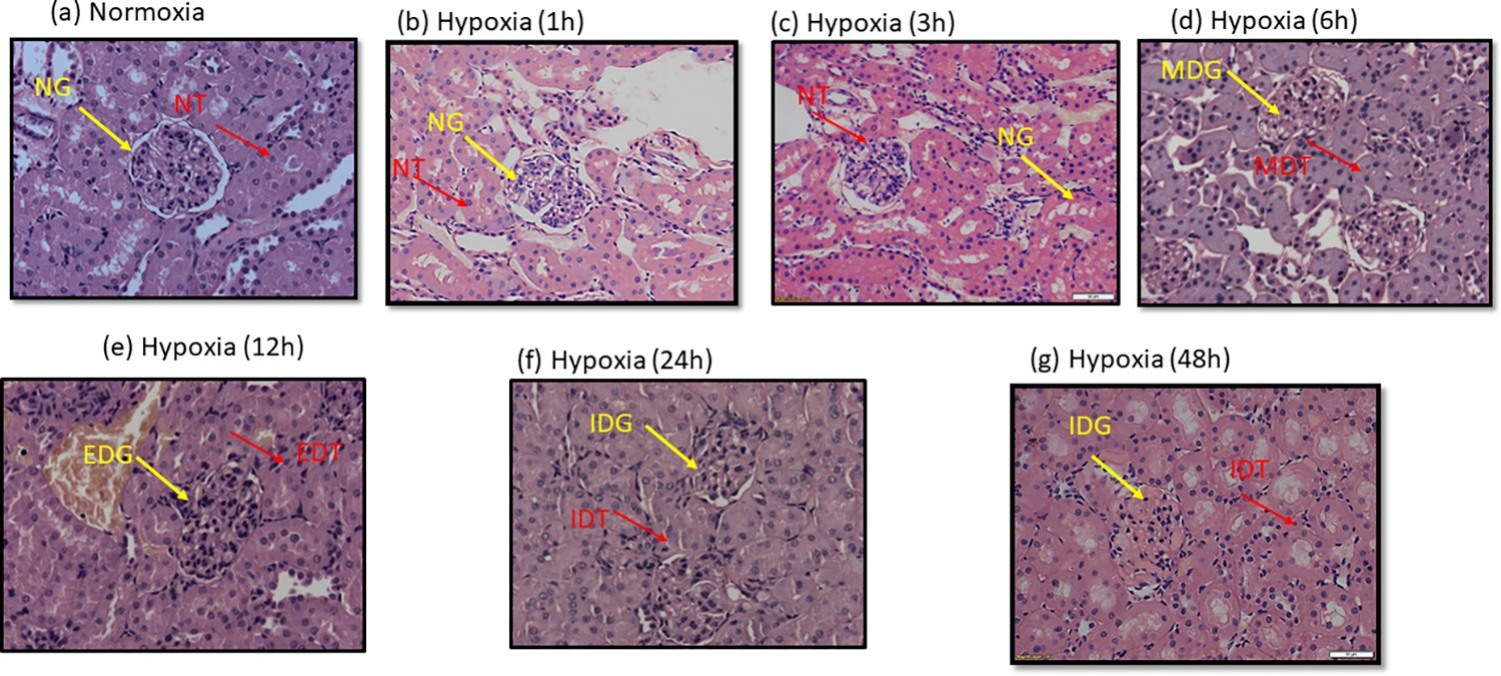
Histopathological images representing the degeneration of renal tubules, glomerulus and Bowman’s capsule, swelling of renal tubules in rats exposed to hypobaric hypoxia for different time durations (0h, 1h, 3h, 6h, 12h, 24h and 48h) at 7620 m (25,000 ft.), 25 ± 2°C (a) Photomicrograph (20X) section of kidney from normoxia control group representing intact normal glomerulus (NG) and Bowman’s capsule with normal tubules (NT) structure without fluid accumulation (b) Photomicrograph (20X) section of kidney from 1h hypobaric hypoxia exposed rats showed unmodified kidney conformation of NG and NT (c) Photomicrograph (20X) section of kidney from group exposed to hypobaric hypoxia (3h) exhibited no difference in kidney configuration with NG and NT (d) Photomicrograph (20X) section of kidney from hypobaric hypoxia exposure for 6h, revealed mild degeneration of glomerulus and Bowman’s capsule (MDG), Mild degeneration of renal tubules (MDT) with mild hypertrophy followed by ballooning of tubular epithelial cells (e) Kidney sections (20X) of hypobaric hypoxia exposed animals for 12h duration had demonstrated the increased tubular cell ballooning, excessive degeneration of glomerulus (EDG), swelling of renal tubules, mesangial proliferation, with large spaces in between the Bowman’s capsular glomeruli along with congested vessels. (f) Photomicrograph (20X) section of kidney from 24h hypobaric hypoxia exposed group manifested the increased degeneration of glomerulus (IDG), increased degeneration of renal tubules (IRT) and (g) a (20X) photomicrograph section of kidney from 24h of hypobaric hypoxia exposed group demonstrated the IDG and IDT. **NG**: Normal glomerulus and Bowman’s capsule, **NT**: Normal renal tubules, **MDG:** Mild degeneration of glomerulus and Bowman’s capsule, **MDT:** Mild degeneration of renal tubules, **EDG:** Excessive degeneration of glomerulus and Bowman’s capsule, **EDT:** Excessive degeneration and swelling in renal tubules **IDG:** Increased degeneration of glomerulus, **IDT:** Increased degeneration of renal tubules.

Histopathology of renal tissue of hypobaric hypoxia exposed rats at 0h, 1h, 3h, 6h, 12h, 24h and 48h of intervals is shown in Fig 5A–5G. Exposure at 1h and 3h of hypobaric hypoxia showed unmodified histopathological changes like intact glomerular and Bowman’s capsule with no tubular degeneration and fluid accumulation in kidney tissue of rats as compared to normoxia control (Fig 5A–5C). However, as the duration of exposure was raised to 6h, mild hypertrophy and ballooning of tubular epithelial cells was started appearing. Heterogeneous patchy regions with tubular rarefaction along with medullary tubular cell atrophy was also observed in kidney sections of these rats (Fig 5D). Whereas, 12h of hypobaric hypoxia exposure, in addition to the characteristic signs of tubular cell ballooning seen in 6h of hypobaric hypoxia exposure, further showed excessive degeneration of glomerulus in the renal cortex, excessive degeneration with swelling of renal tubules along with increased mesangial proliferation followed by decreased Bowman’s capsular space in few glomeruli. At these places, vessels appeared to be narrow and congested. However, further, increase in hypobaric hypoxia exposure duration up to 24h and then also up to 48h, resulted into more or less similar intensity of damage compared to 12 h of hypoxia exposure duration. This indicate that, the extent of kidney damage that could take place under hypobaric hypoxia exposure has reached to peak from 12h of hypobaric hypoxia exposure and after that it remained constant up to 48 h of hypobaric hypoxia exposure. Therefore, in this acute phase study, 12h of hypobaric hypoxia exposure was considered as the optimum time at which the maximum renal damage occurred in kidneys of rats.

### Phase II: Quercetin dose optimization study

#### Effect of different doses of quercetin on oxidative stress

**Fig 6:**
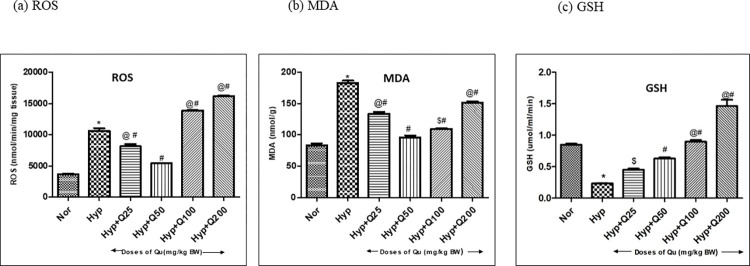
Effect of different doses of quercetin (25mg, 50mg, 100mg and 200mg/Kg BW) on (a) Reactive Oxygen Species (ROS) (b) Malondialdehyde (MDA) and (c) Reduced Glutathione (GSH) levels in kidneys of rats exposed to hypobaric hypoxia at 7,620m for 12h at 25 ± 2°C. Values are mean ± SD (n = 6). (a) ROS: *p<0.001 Norm v/s Hypo (12h); #p<0.001 Hypo v/s Hypo+ Q25mg, Hypo + Q50mg, Hypo+ Q100mg, Hypo+Q200mg; @p<0.001 Hypo+ Q50mg v/s Hypo+Q25mg, Hypo+ Q100mg, Hypo+Q200mg (b) MDA: *p<0.001 Norm v/s Hypobaric Hypo (12h); #p<0.001 Hypo v/s Hypo+ Q 25mg, Hypo + Q50mg, Hypo+ Q100mg, Hypo+Q200mg; @p<0.001 Hypo+ Q50mg v/s Hypo+ Q25mg, Hypo+Q200mg, $p<0.05 Hypo + Q50mg v/s Hypo+ Q100mg (c) GSH: *p<0.001 Norm v/s Hypo (12h); $p<0.05 Hypo v/s Hypo+ Q 25mg, #p<0.001 Hypo v/s, Hypo + Q50mg, Hypo+ Q100mg, Hypo+Q200mg, +p<0.01 Hypo+ Q50mg v/s Hypo+ Q100mg; @p<0.001 Hypo+ Q50mg v/s Hypo+ Q200mg. Norm-Normoxia control, Hypo- Hypobaric hypoxia exposure of 12h and Q 25, Q50, Q100 and Q200 mg- Quercetin doses @ 25, 50, 100 and 200 mg/Kg BW.

*Reactive Oxygen Species (ROS)*. ROS generation in rats administered with different doses (i.e. 25, 50, 100, 200mg/kg BW) of quercetin 1h prior to hypobaric hypoxia exposure is depicted in Fig 6A. Hypobaric hypoxia exposure resulted in to increased (2.9-fold ↑) ROS generation in 12h hypoxia exposed rats compared to Normoxia control. Quercetin supplementation (25 and 50mg/kg BW) showed effective reduction in ROS production (p<0.001) compared to control (Hypoxia). Although, rats fed with 25mg/kg BW quercetin reduced the ROS levels significantly (1.3 fold ↓), but the reduction was not very much appreciable when compared with 50mg/kg BW quercetin fed hypoxia exposed rats (2 fold ↓). However, as the doses increased beyond 50 mg/kg BW (i.e., 100 and 200mg/kg BW) a significant elevation in reactive oxygen species levels were observed (1.3-fold↑ and 1.5-fold↑ respectively) compared to hypoxia control indicating towards the pro-oxidant property of quercetin at these doses.

*Lipid peroxidation (MDA)*. Almost similar inclination (as that of ROS) was illustrated by MDA as well, wherein hypobaric hypoxia exposure resulted in to increased (2.2-fold↑) MDA production compared to normoxia control. All the tested doses showed reduction in kidney MDA levels as compared to the hypoxia control. However, administration of 50mg/kg BW of quercetin manifested the lowest levels of significant reduction (2-fold↓) in lipid peroxidation (Fig 6B) in kidney homogenates of rats compared to all the tested doses. Whereas, doses beyond 50 mg/kg BW (i.e. 100 and 200mg/kg BW) were observed to have deleterious effects as the MDA levels exhibit a significant (1.1-fold↑ and 1.5-fold↑, respectively) increment when compared with 50mg/kg BW quercetin administered hypobaric hypoxia exposed groups (Fig 6B).

*Reduced Glutathione (GSH)*. Hypoxia exposure (12h) significantly reduced (4-fold↓) the GSH levels in kidneys of rats compared to normoxia control. There was a consistent and significant increase in kidney GSH levels were observed in all the doses tested under hypobaric hypoxia exposure as compared to Hypoxia control. 50mg/kg BW quercetin pretreatment 1h prior to hypobaric hypoxia exposure significantly enhanced the antioxidant GSH (3 fold ↑) levels in kidneys that exhibited a significant downfall (4-fold↓) due to hypoxia exposure (12h). However, administration of 25 mg/kg BW failed to enhance the GSH activity efficiently when compared with 50mg/kg BW fed rats. Moreover, supplementation of quercetin beyond 50mg/kg BW dose (i.e. 100 and 200 mg/kg BW) further enhanced the kidney GSH levels compared to hypoxia control (Fig 6C).

#### Effect of different doses of quercetin on renal function parameters under hypobaric hypoxia exposure

**Fig 7:**
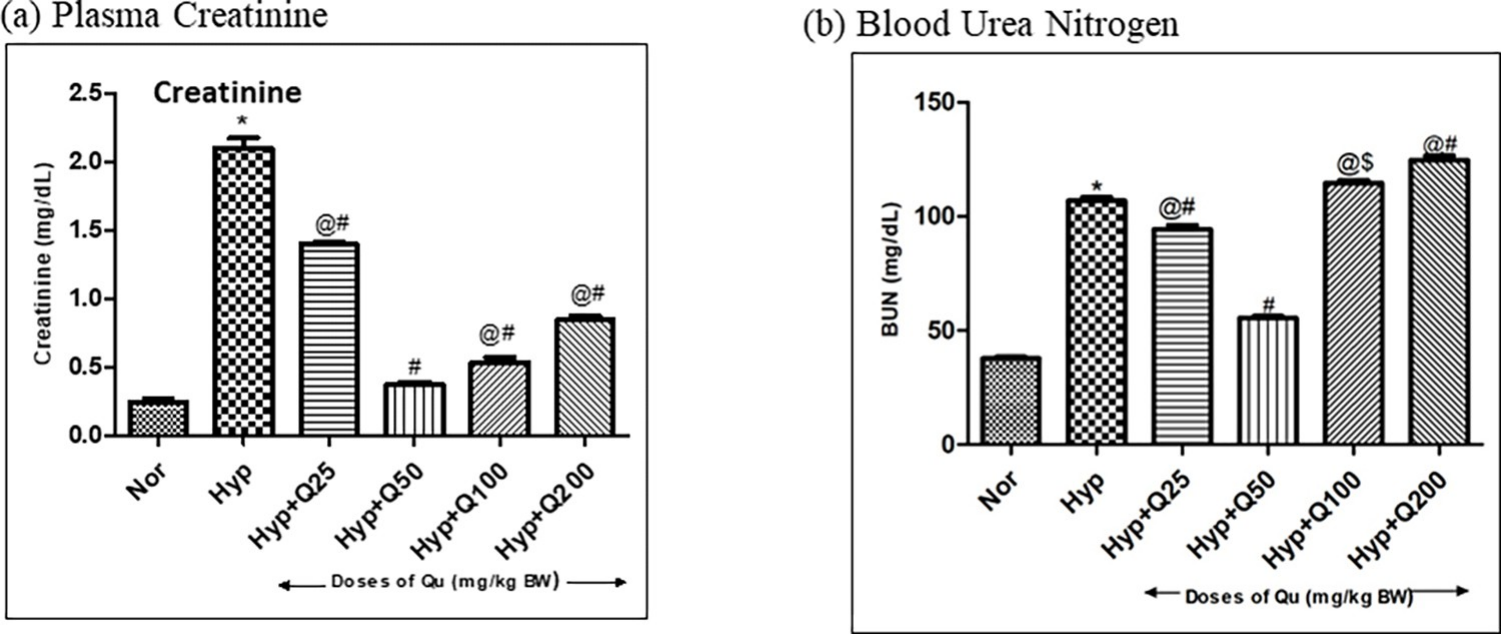
Effect of different doses of quercetin (25mg, 50mg, 100mg, 200mg/Kg BW) supplementation to rats exposed to 7,620m for 12h at 25 ± 2°c on (a) Plasma Creatinine levels *p< 0.001 Norm v/s Hypo; #p< 0.001 Hypo v/s Hypo+Q25mg, Hypo+ Q50mg, Hypo+ Q100mg and Hypo+ Q200mg; @p< 0.001 Hypo+ Q50mg v/s Hypo+ Q25mg, Hypo+ Q100mg, and Hypo+ Q200mg (b) Plasma BUN levels *p< 0.001 Norm v/s Hypo; #p< 0.001 Hypo v/s Hypo+ Q25mg, Hypo+ Q50mg and Hypo+ Q200mg; $p< 0.1 Hypo v/s Hypo+Q100mg, @p< 0.001 Hypo+ Q50mg v/s Hypo+ Q25mg, Hypo+ Q100mg, Hypo+ Q200 mg) levels in rats exposed to hypoxia. Norm-Normoxia control, Hypo- Hypobaric hypoxia exposure of 12h and Q25, Q 100 and Q200 mg- Quercetin @ 25, 50, 100 and 200 mg/Kg BW.

As a result of hypobaric hypoxia exposure (12h), plasma creatinine and BUN levels exhibited a significant elevation (i.e., 8.8-fold ↑and 3.1-fold↑ respectively) when compared with normoxia control. However, reduction in plasma creatinine and BUN (7-fold ↓ and 2.2-fold ↓ respectively) levels were found in quercetin (50mg/kg BW) supplemented group as compared to the hypoxia control rats. Although, a significant (P<0.05) reduction in creatinine was observed in 25mg/kg BW quercetin supplementation as well, however, that does not appear to be efficiently competent when compared with 50mg/kg BW quercetin supplemented rats (Fig 7A). Similarly, Quercetin (50mg/kg BW) preconditioning managed to eliminate BUN efficiently. A downfall in its value was observed in quercetin administered groups (25 mg/kg BW) however, that does not appear to be efficient when compared with reduction attained with 50mg/kg BW dose of quercetin (Fig 7B). Moreover, quercetin supplementation at 100mg/kg BW and 200mg/kg BW exhibited elevated levels of plasma creatinine as compared to hypoxia control. Further, we have noticed that, BUN levels were significantly very high in 100mg/kg BW and 200mg/kg BW of quercetin supplementation as compared to 50mg/kg BW supplemented rats that indicate towards possibility of deleterious effects of quercetin more than 50mg/kg BW dosage, as these two doses (100 and 200 mg/kg BW) showed enhanced ROS production (Fig 6A) as well.

### Phase III: Efficacy of quercetin in elimination of oxidative stress and renal injury

#### Effect of 50mg/kg BW dose of quercetin on oxidative stress in SD rats exposed to 12h of hypobaric hypoxia

**Fig 8:**
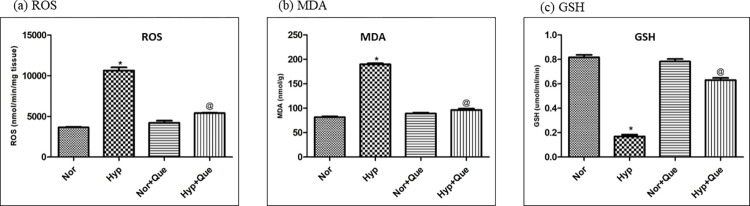
Effect of quercetin supplementation (50mg/Kg BW) in kidneys of rats exposed to 7,620m for 12h at 25 ± 2°c (a) Reactive Oxygen Species (ROS) (b) Malondialdehyde (MDA) and (c) Reduced Glutathione (GSH) generation in kidneys of rats exposed to hypobaric hypoxia at 7,620m for 12h at 25 ± 2°C. *p< 0.001 Norm v/s Hypo 12h; @ p< 0.001 Hypo v/s Hypo+ Que. Values are mean ± SD (n = 6). Norm-Normoxia control, Hypo–Hypobaric Hypoxia (12h); Que- Quercetin (50mg/kg BW).

The efficacy of quercetin in quenching the ROS generated due to low oxygen availability is demonstrated in Fig 8A. Hypobaric hypoxia exposure (12h) manifested 3-fold ↑ increase in ROS generation in kidneys of rats as compared to normoxia control. However, administration of 50mg/kg BW quercetin prior to hypoxia exposure reduced the ROS production (2-fold ↓) when compared with hypoxia control group (12h). Further, an increase (2.5 fold ↑) in MDA production of hypobaric hypoxia exposed rats (as compared to control group) was reduced to normal levels (2-fold decline) when compared with 50mg/kg BW quercetin administered hypobaric hypoxia exposed rats (p<0.05). Fig 8B illustrates the efficiency of quercetin in prevention of lipid peroxidation in kidneys of rats. Additionally, a significant (p<0.001) upregulation of GSH in quercetin preconditioned hypobaric hypoxia exposed rats was also observed as compared to hypobaric hypoxia (12h) control rats that was significantly declined (4-fold ↓) due to 12h of hypobaric hypoxia exposure (Fig 8C). However, insignificant difference was observed in ROS, MDA and GSH levels in kidneys of rats supplemented with quercetin under normoxia as compared to normoxia control rats (Fig 8A–8C).

#### Effect of 50mg/kg BW dose of quercetin on renal function parameters in rats exposed to 12h of hypoxia

**Fig 9:**
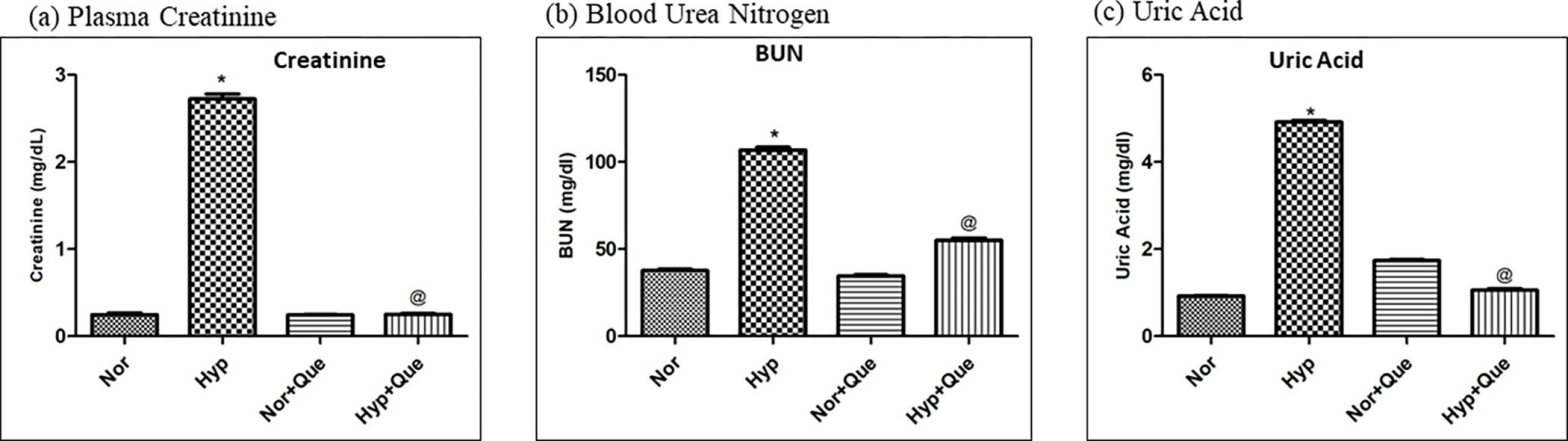
Effect of quercetin supplementation (50mg/Kg BW) in kidneys of rats exposed to 7,620m for 12h at 25 ± 2°C (a) Plasma Creatinine (b) Plasma BUN (c) Plasma Uric Acid levels *p< 0.001 Norm v/s Hypo 12h; @ p< 0.001 Hypo v/s Hypo+ Que. Norm-Normoxia control, Hypo 12h- hypobaric hypoxia exposure for 12h, Norm+ Que-Quercetin administered to normoxia control, Hypo+ Que- Quercetin administered hypoxia exposure of 12h. Norm- Normoxia, Hypo-Hypoxia; Que- Quercetin (50mg/kg BW).

The hypoxia (12h) exposed group showed a marked increase (P<0.001) in plasma creatinine, BUN and uric acid levels in rats compared to control (Normoxia). However, a significant reduction in their levels were observed in hypoxia exposed rats supplemented with 50mg/kg BW quercetin (1h prior to hypoxia exposure) in comparison to hypobaric hypoxia control group (12 h) (p<0.05). Further, it was noticed that, rats receiving quercetin (50mg/kg BW) under normoxia exhibited unmodified creatinine, BUN and uric acid levels in plasma of rats compared to normoxia control (Fig 9A–9C).

#### Effect of 50mg/kg BW dose of quercetin on lactate dehydrogenase (LDH) in kidneys of rats exposed to 12h of hypoxia

**Fig 10:**
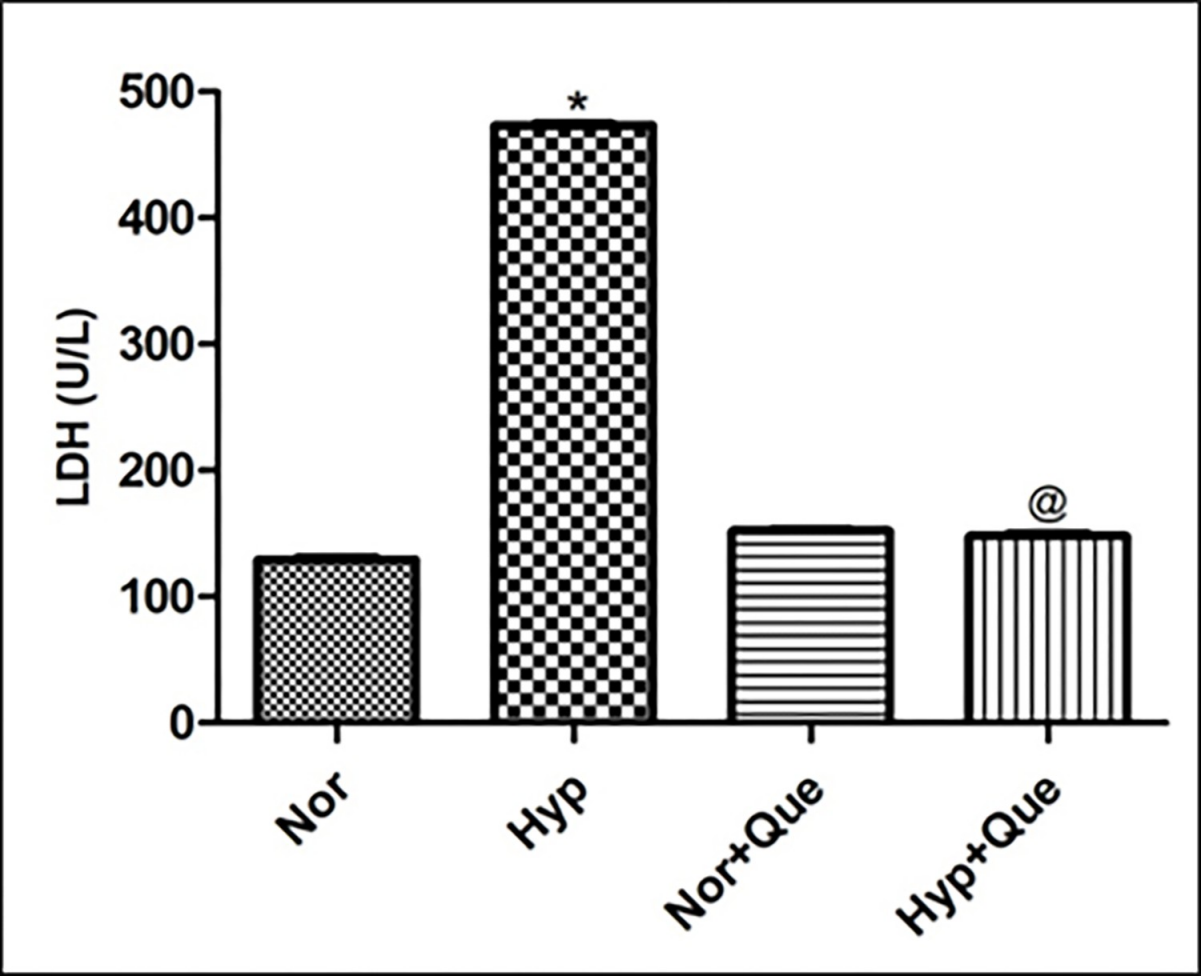
Effect of quercetin on lactate dehydrogenase (LDH) (renal tissue injury) in rats supplemented with 50mg/kg BW quercetin prior to hypoxia exposure of 12h at 7,620m, 25 ± 2°Values are mean ± SD (n = 6). *p< 0.001 Norm v/s Hypo 12h; @ p< 0.001 Hypo v/s Hypo+ Que. Norm-Normoxia control, Hypo–Hypoxia exposure of (12h), Que- Quercetin (50mg/kg BW).

As expected hypoxia exposed animals showed elevated LDH levels in kidney homogenate of rats compared to normoxia control. Whereas, quercetin (50mg/kg BW) preconditioning reduced the hypoxia induced renal injury (LDH levels) (by approx. 4 folds ↓) over control (Hypoxia 12h) (Fig 10). However, Normoxia animals supplemented with Quercetin did not show any significant difference in LDH levels in kidneys of rats in comparison to normoxia control rats (Fig 10).

#### Effect of 50mg/kg BW dose of quercetin on protein expression of HIF-1 α and VEGF

**Fig 11:**
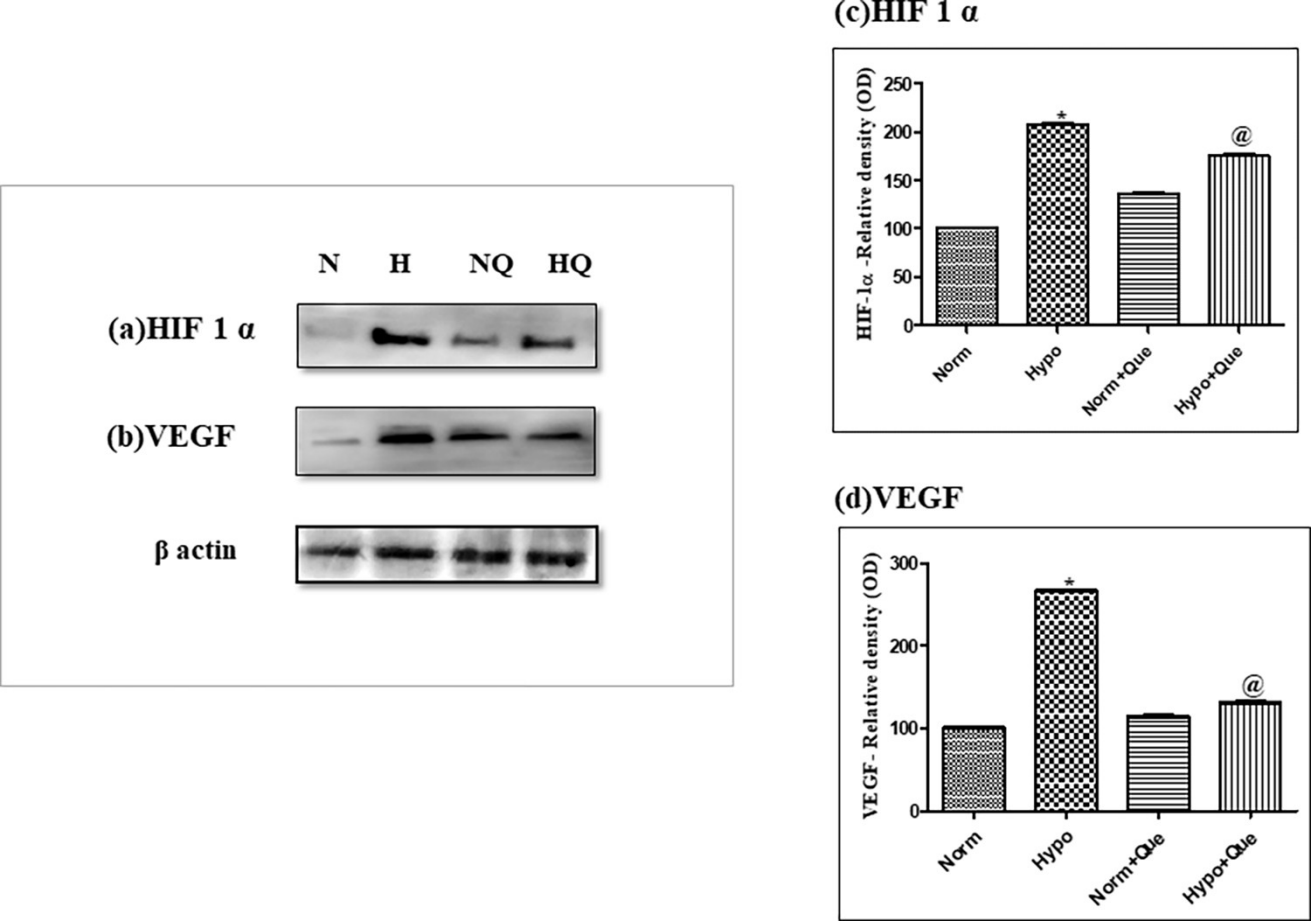
Effect of quercetin on protein expressions of (a) HIF-1α and (b) VEGF levels in rats supplemented with 50mg/kg BW quercetin 1h prior to hypobaric hypoxia exposure of 12h at 7,620m, 25 ± 2°C. (c) Densitometry analysis of HIF-1 α *p< 0.001 Norm v/s Hypo 12h; @ p< 0.001 Hypo v/s Hypo+ Que. (d) Densitometry analysis of VEGF: *p< 0.001 Norm v/s Hypo 12h; @ p< 0.001 Hypo v/s Hypo+ Que. N = Normoxia, H = Hypoxia, NQ = Normoxia + Quercetin (50mg/kg BW), HQ = Hypoxia + Quercetin (50mg/kg BW).

Hypobaric hypoxia exposure appreciably stabilized the HIF-1 α protein expression in kidneys of rats as compared to normoxia control. One of the genes regulated by HIF-1 α is VEGF, which was found to be upregulated under hypobaric hypoxia exposure compared to normoxia control. 50mg/kg BW quercetin administration stabilized the HIF-1 α protein expression and reduced the VEGF protein expression levels in quercetin treated group as compared to hypobaric hypoxia exposed groups (Fig 11A and 11B respectively). The densitometry analyses of these proteins were expressed in adjacent to their respective figures (Fig 11C for HIF-1 α and Fig 11D for VEGF).

#### Histopathological image analysis

**Fig 12:**
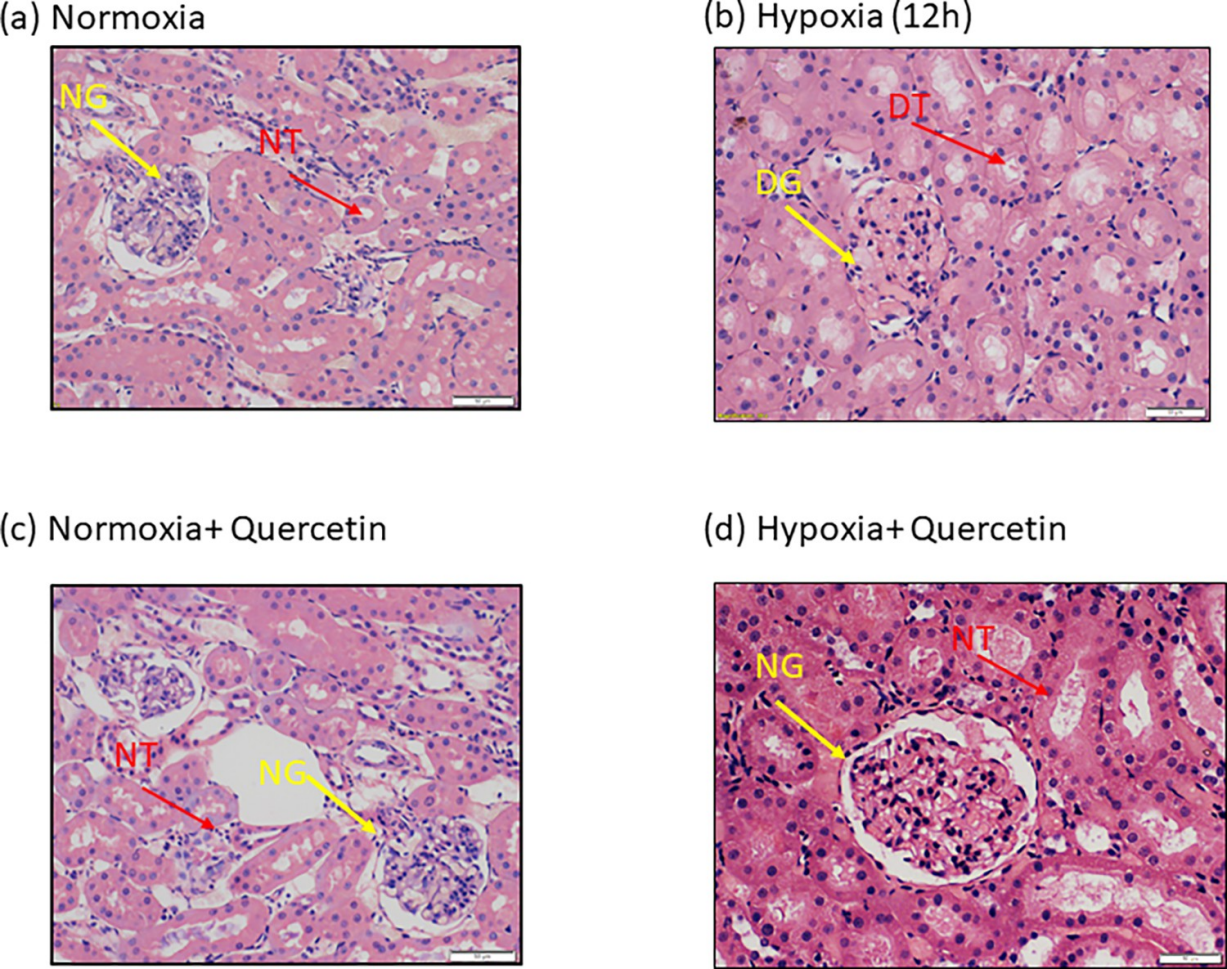
(a-d) Renal pictomicrographs images depicting the degeneration of renal tubules, glomerulus and Bowman’s capsule, swelling of renal tubules in rats exposed to hypobaric hypoxia at 7,620m, 25 ± 2°C for 12h. (a) Photomicrograph (20X) section of kidney from Normoxia control group representing intact normal glomerulus (NG) and Bowman’s capsule with normal tubules (NT) configuration (b) Kidney sections (20X) of hypobaric hypoxia exposed animals for 12h duration had demonstrated the excessive degeneration of glomerulus with loss in integrity of Bowman’s capsule (EDG), Excessive ballooning of tubular cells, along with swelling of renal tubules (EDT) and mesangial proliferation. These animals (12h) also showed large spaces in between the Bowman’s capsular glomeruli and congestion of vessels (c) Photomicrograph (20X) sections of kidney from normoxia animals receiving Quercetin showed no noticeable changes as compared to normoxia control and (d) whereas rats exposed to hypobaric hypoxia (12h) and supplemented with Quercetin showed no structural changes in glomerulus and Bowman’s capsule along with clear tubular structures. **EDG:** Excessive degeneration of glomerulus and Bowman’s capsule, **EDT:** Excessive degeneration and swelling in renal tubules. **NG**: Normal intact glomerulus and Bowman’s capsule, **NT**: Normal renal tubules.

The tissue sections of normoxia control rats were seen to possess intact and complete Bowman’s capsule with typical normal renal tubules configurations with appropriate interstitial space (Fig 12A). However, kidneys sections of hypobaric hypoxia exposed rats for 12h exhibited Excessive degeneration of glomerulus and Bowman’s capsule (EDG) with excessive degeneration with swollen renal tubules (EDT) (Fig 12B). Further, reduction in glomerular degeneration and tubular dystrophy in quercetin treated hypoxia exposed group (Fig 12D) was detected as compared to hypoxia exposed group (Fig 12B). Ballooning in renal tubules was absent in quercetin treated group in comparison with hypoxia exposed groups for 12 h hypoxia exposure duration (Fig 12B). However, no such defect was observed in quercetin treated normoxia group as compared to normoxia control group of rats (Fig 12C).

## Discussion

**Fig 13:**
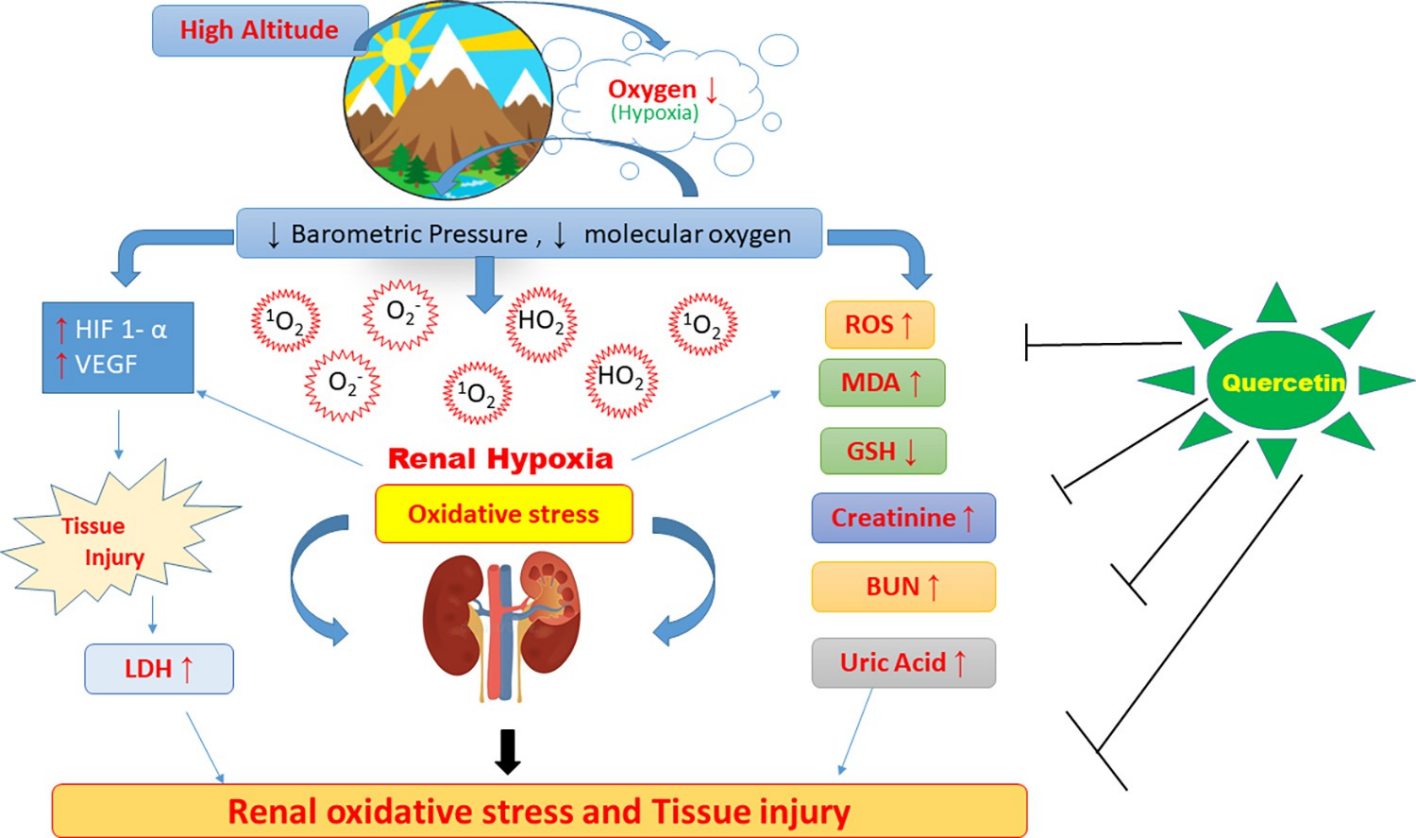
Schematic representation of altered renal functions with elevated oxidative stress under hypobaric hypoxia and plausible mechanism of their prevention by quercetin administration in rats. ROS—Reactive oxygen species, MDA- Malondialdehyde, GSH- reduced glutathione, BUN—Blood urea nitrogen, HIF-1α—Hypoxia inducible factor 1 alpha, VEGF-Vascular Endothelial Growth Factor, LDH- lactate dehydrogenase. ↑-Upregulation, ↓- Downregulation, ⊥- Attenuation.

The initial renal physiological alteration at high altitude regions is a complex process that is not yet fully explored. In this study, we intended to understand the hypoxia induced renal oxidative stress and how renal oxygen homeostasis and metabolism is defended during different time durations of hypoxia exposure at simulated high altitude. Further, efforts were made to prevent hypoxia induced deleterious damage in the kidneys of rats by prophylaxis with a flavonoid, quercetin. The present study reports that the exposure of animals to hypoxia for different durations enhanced the ROS generation, Lipid peroxidation (MDA production), and LDH levels and reduced the GSH levels in kidneys followed by increased Creatinine, BUN, and uric acid levels in plasma along with stabilized HIF-1 alpha and increased VEGF protein in kidneys of rats exposed to hypobaric hypoxia time dependently from 1h to 48h compared to control. Hypoxia exposure of 12h inflicted maximum measurable deteriorating renal oxidative and metabolic effects in the kidneys of rats thus, it was regarded as the optimum time duration of hypobaric hypoxia exposure at which maximum renal damage would occur. Similarly, in the present study, the dose dependent studies conferred the optimum dose of Quercetin supplementation at which the minimum renal injury obtained was at 50 mg/kg BW compared to other tested doses at 12h of hypobaric hypoxia exposure. Finally, we have reported that, the rats receiving 50mg/kg BW of quercetin 1 hour prior to 12h of hypobaric hypoxia exposure significantly exhibited a reduction in hypoxia induced oxidative stress and renal injury in rats which is schematically represented in Fig 13 and is sequentially discussed in the ensuing paragraphs.

Chhabra et al. (2018) have stated that, an altered redox pathway plays a significant role in renal function impairment in rats under hypobaric hypoxia [29]. Thereupon, the study presented here reports a marginal and insignificant increment in ROS production starting from 1h of hypoxia exposure that increases gradually and significantly as the duration of exposure rises from 3h to 48h. Unlike ROS, the production of malondialdehyde formed as a byproduct of peroxidation of polyunsaturated fatty acids in the cells, exhibited a significant increase right from 1 hour of hypobaric hypoxia exposure to 48h (p<0.001) of exposure. This indicates the vulnerability of kidneys to hypoxic conditions due to the presence of polyunsaturated fatty acids. Additionally, GSH levels were observed to be declined from 1h-3h of hypobaric hypoxia exposure, however major noteworthy downfall was observed in 6h to 48h of hypobaric hypoxia exposure compared to normoxia control. Subsequently, renal functions (measured in terms of accumulation of creatinine, blood urea nitrogen, and uric acid in plasma) also experienced a significant downfall due to compromised oxygen content which prevailed at hypobaric hypoxia conditions. However, the study speculated an interesting phenomenon that even though an upsurge was observed in ROS production from 1h of hypobaric hypoxia exposure but creatinine and BUN retention in plasma occurred from 3h and 6h of hypobaric hypoxia exposure. This indicates that kidneys respond to hypoxia induced oxidative stress from 1h onwards, however, its metabolic effect was initiated from 6h and reached to maximum by 12h of hypoxia exposure, indicating the sustainability of kidney up to 6h of hypobaric hypoxia stress, later the kidneys are much vulnerable to oxidative stress. These findings were further correlated and confirmed by histopathological studies.

‘High altitude renal syndrome’ (HARS) is the term associated with renal complications occurred due to diminished oxygen availability at high altitudes. The related conflictions are reported in individuals ascending to approximately 3650m from 50m within 4–6 hours of ascent [30]. Unlike other tissues, partial pressure of oxygen in kidneys (renal oxygenation) is tightly controlled and does not always in direct proportion with renal blood flow. Steele et al. (2020) have reported that a decrease in renal oxygen delivery (RDO_2_) was observed in subjects after 12h of rapid ascent from sea level to Cerro de Pasco, Peru, 4,330 m. However, after 7 days of acclimatization at 4,330m of altitude RDO_2_ was found to be stabilized via increased renal oxygen delivery and blood flow [31]. Limited but time-based data available, predominantly evaluates chronic hypoxia exposure to rats and reported considerable deviations after 7 days of hypoxia exposure [29, 32]. As long as our knowledge is concerned, this article is the first study to demonstrate the consequences of acute hypobaric hypoxia exposure at different intervals of time durations on the renal system of the Sprague Dawley rat model and its amelioration by Quercetin prophylaxis.

The maximum renal damage that was observed in the present study was due to compromised low oxygen availability after 12h of simulated hypobaric hypoxia exposure in rats. At this hour, renal impairment, reflected by creatinine, blood urea nitrogen & uric acid levels were maximum along with tissue injury as determined by lactate dehydrogenase production by damaged renal cells due to lack of oxygen. Systemic conflictions associated with blood urea nitrogen, creatinine & uric acid, mark the abnormal functioning of kidneys at this (12h) hour of hypobaric hypoxia exposure. Further, the histopathological assessment showed excessive ‘ballooning’ of renal tubules occupying interstitial space with excessive disintegration of Bowman’s capsule prominently initiated from 12h of hypobaric hypoxia exposure. However, structural alterations remained constant with increasing time duration from 12h to 48h as compared to the normoxia group. It indicates that as the exposure time of hypobaric hypoxia duration increased the renal damage also increased, the peak being at 12h of hypoxia exposure.

The dimerization of HIF-1 α with HIF-1 β leads to their binding with hypoxia response elements (HRE) of various HIF regulated genes [33–35]. Likewise, its binding to the hypoxia response element (HRE) of VEGF promoter region that includes cis-acting DNA elements recognized by multiple trans activators, results in its transactivation and further leads to angiogenesis [36, 37], tissue perfusion, and recovery from hypoxia-induced tissue damage [38]. Protein expression analysis showed continuous upregulation of HIF-1 α expression with increasing time duration from 3h to 48h as compared to the control group. However, HIF- 1α expression attained a stable expression as the time reaches 12h to 48h. As a master regulatory gene, HIF- 1α enhances the production of several proteins to combat the growing need for oxygen under hypoxic conditions. One among them is vascular endothelial growth factor (VEGF), essential for the formation of new blood vessels required for maximum oxygen delivery at tissue sites was found to increase from 12h to 48h over normoxia control. Thus, we confirmed that 12h of hypoxia exposure provided maximum renal damage in rats.

Quercetin, a phytoflavanoid, present in grapes, berries, cherries, onion, etc. exhibits anti-oxidative and anti-inflammatory properties. Quercetin, being a well-established antioxidant and anti-inflammatory molecule manifests its properties via quenching reactive oxygen species, improving GSH production, inhibiting associated enzymes, and modulating several signal transduction pathways [39]. However, its efficacy in improving impaired renal functions and hypobaric hypoxia associated renal damage is not much known. Therefore, we tried to explore the most proficient dose of quercetin in preventing renal alterations under hypobaric hypoxia. Among all the tested doses, quercetin at 50mg/kg BW showed highest protective activity in reducing the ROS, MDA and maintained the kidney parameters at controlled levels. However, doses beyond 50 mg/kg BW enhanced the above mentioned parameters which indicate towards the pro-oxidant nature of this flavonoid. Numerous researchers [40–42] have explored the pro-oxidant property of Quercetin supplemented at higher concentrations. A study by Spencer et al., (2003) demonstrated that the protective role of quercetin at 10μM of concentration against dermal fibroblast cells’ damage caused by oxidative stress, whereas cytotoxicity was encountered in these cells, when quercetin dose was increased up to 30μM indicating that, Quercetin shows dual the effect as both pro and anti-oxidant properties depending upon the dose used [43]. In relation to these findings, Vieira-Frez et al. (2020) have reported that quercetin at higher doses (100mg-1) exerted pro-oxidant property by aggravating the ROS production and further showed harmful effects in diabetic rats and at the same time, low dose administration of quercetin (10 mg/kg-1) gave rise to antioxidant property consequently protected the rats from oxidative stress [44]. It is therefore very much important to standardize the dose escalations before using quercetin in preclinical and clinical studies. Similarly, in the present study, we also noticed that as the concentration of the quercetin dose increased from 50 mg/kg BW to 100 and 200 mg/kg BW, the increased GSH levels were not able to quench the enhanced ROS generated under hypobaric hypoxia condition, indicating the pro-oxidant property of quercetin given at higher concentrations. This is probably a consequence of auto-oxidation of the quercetin at higher doses of administration [45, 46]. Therefore, these studies revealed that 50 mg quercetin /kg BW is the optimum dose at which reduced ROS production and MDA levels with enhanced GSH levels were obtained in rats under hypobaric hypoxia.

In a recent study Chiş et al., (2018) demonstrated the protective effects of quercetin in heart tissues towards hypoxia induced oxidative/nitrosative stress by increasing SOD and CAT and reducing the MDA and protein carbonyl [47] and also increased the endogenous enzymatic antioxidant activity in the brain [48, 49]. It is factual that; Quercetin being lipophilic in nature may therefore penetrate deeper into the cell membrane layers to scavenge the lipid radicals. Quercetin enhances the body’s antioxidant capacity also by regulating the levels of GSH. It is reported that, the 1% quercetin diet induces the expression of GPx, CAT, and SOD with a reduction in lipid peroxidation in the liver and epididymal adipose tissue [50, 51]. A study exploring the efficacy of quercetin in mitigating oxidative stress induced damage due to hypoxia in cardiomyocytes reported its beneficial effects by regulating ER function, mitochondrial quality control, reducing capillary fragility, etc. [52] and a better scavenger of O_2_- than NO under increased O_2_- concentrations in smooth muscles of blood vessels [53].

Hypoxia induced VEGF upregulation amplifies vascular angiogenesis to oxygen-deprived organs. However, increased vascular permeability leading to renal angiogenesis has an injurious role [54]. In the present study, Quercetin supplementation stabilized the HIF-1 alpha transcription activity with subsequent reduction in VEGF expression for better acclimatization at high altitudes. The results obtained here are in accordance with the results reported earlier by Ansó et al., (2010), Alshanwani et al., (2020) [55, 56]. Beneficial effects of quercetin were also observed in protection against hypoxia induced renal corpuscle and tubular degeneration with the enhanced structural arrangement in flavonoid supplemented rats [57]. Similar results were reported earlier by Liu et al. [57]. Further, the administration of quercetin has renal protective effects that result in a significant decline in plasma creatinine, BUN, and LDH levels. The results obtained in the present study were in correspondence with the results reported earlier [58, 59]. All these findings clearly indicate the possible potential use of quercetin in clinical applications. A schematic representation of the present study is depicted in Fig 13.

## Conclusion

The findings of this study revealed that acute hypobaric hypoxia exposure of 12h enhanced the oxidative stress that resulted into impaired renal function along with renal tissue damage. Whereas quercetin administration (50mg/kg BW) significantly prevented the hypobaric hypoxia associated renal complications. Being a potent antioxidant, quercetin declined the oxidative stress, stabilized HIF-1α and reduced VEGF protein expression that resulted in to better renal functioning under hypoxia. Thus, present data indicate the protective prophylaxis with quercetin abrogates the possibility of renal injury under hypobaric hypoxia conditions.

## Supporting information

S1 Raw images Full Blot images of figures of Figs 4A–4C and 11A–11C. (PDF)
