# Supplementary material for: Hypobaric hypoxia induced renal injury in rats: Prophylactic amelioration by quercetin supplementation
Source: PLoS One. 2023 Feb 24;18(2):e0279304. doi: 10.1371/journal.pone.0279304 (PMC9955615; doi:10.1371/journal.pone.0279304)
Supplement: S1 Raw images — (PDF) [file pone.0279304.s001.pdf]

Figure 4 (a) HIF-1α

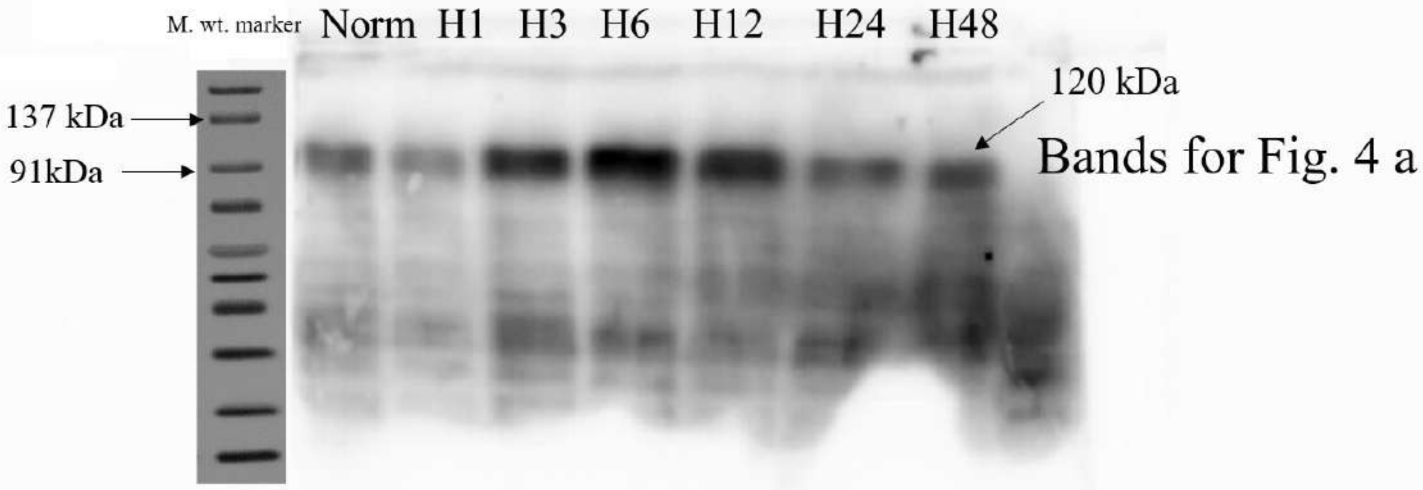

Figure 4 (b) VEGF

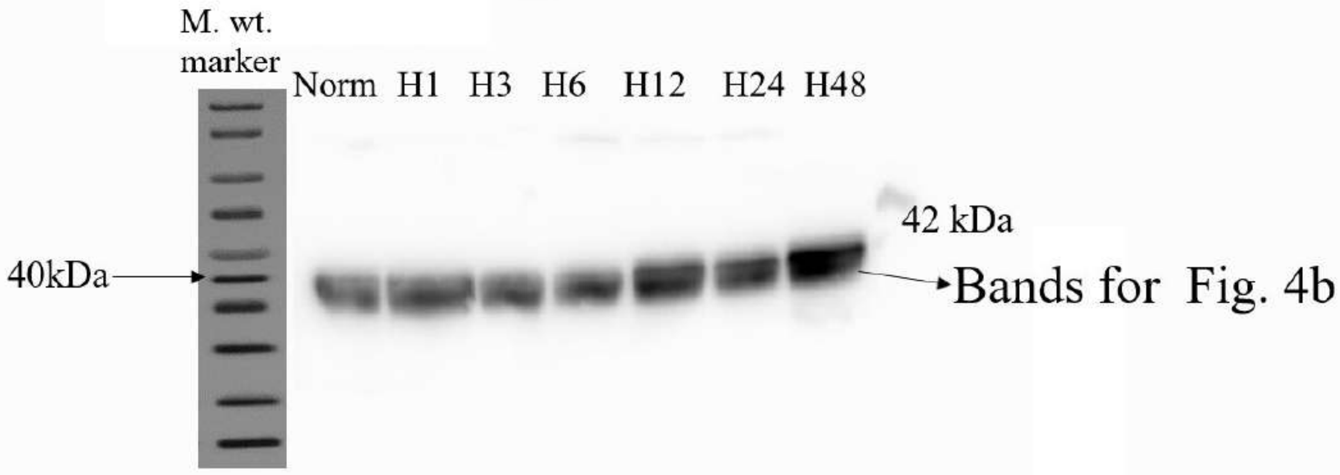

Figure 4 (c) β- actin

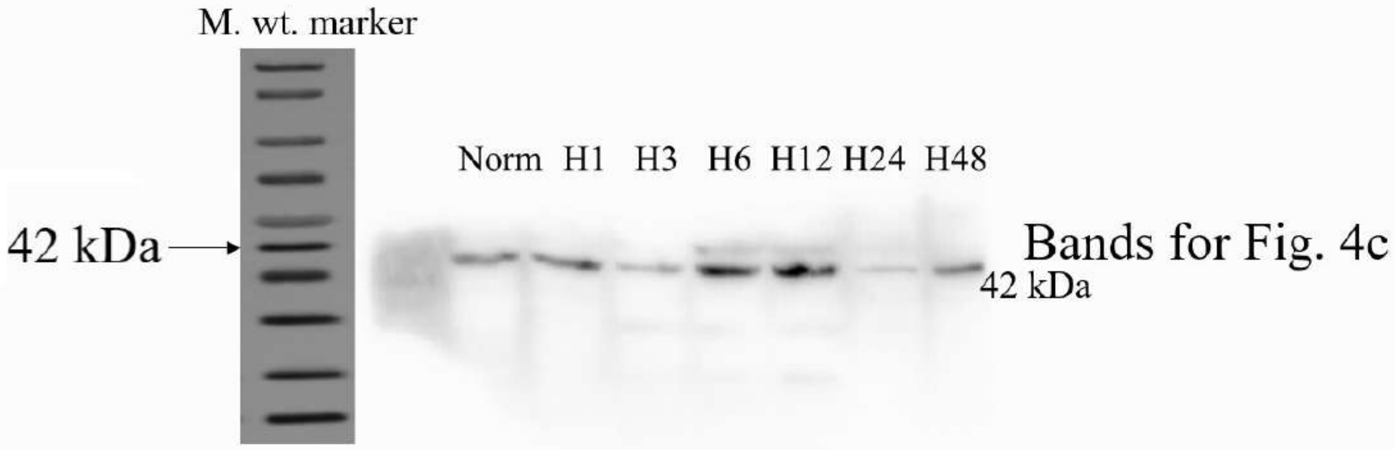

PONE-D-22-19505R1, Hypobaric hypoxia induced renal injury in rats: Prophylactic amelioration by quercetin supplementation

Figure 11 (a) HIF-1α

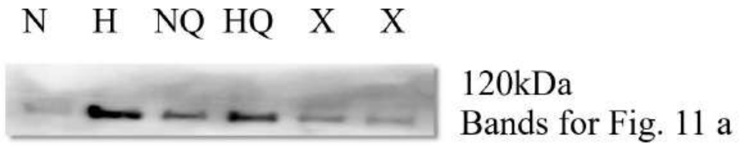

'X' stands for extra bands hence, not to be considered

Figure 11 (b) VEGF

N H NQ HQ X X

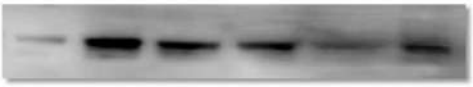

40kDa
Bands for Fig. 11 b

'X' stands for extra bands hence, not to be considered

PONE-D-22-19505R1, Hypobaric hypoxia induced renal injury in rats: Prophylactic amelioration by quercetin supplementation

Figure 11 (c) β- actin

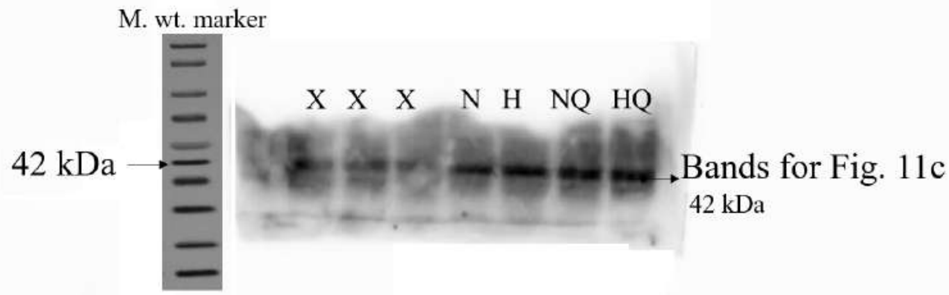

'X' stands for extra bands hence, not to be considered
